# The MacNew Heart Disease Health-Related Quality of Life Questionnaire: A Scandinavian Validation Study

**DOI:** 10.1007/s11205-014-0694-7

**Published:** 2014-07-24

**Authors:** Shan Alphin, Stefan Höfer, Joep Perk, Stig Slørdahl, Ann-Dorthe Olsen Zwisler, Neil Oldridge

**Affiliations:** 1 Department of Cardiology, Holbæk Hospital, Copenhagen, Denmark; 2 Department of Medical Psychology, Innsbruck Medical University, Innsbruck, Austria; 3 Department of Cardiology, Public Health Department, Kalmar County, Oskarshamm, Sweden; 4 Department of Circulation and Medical Imaging, Norwegian University of Science and Technology, Trondheim, Norway; 5 Institute of Public Health, University of Southern Denmark, Copenhagen, Denmark; 6 School of Medicine and Public Health, University of Wisconsin, Milwaukee, WI USA; 7 Aurora Cardiovascular Services, Aurora St. Luke’s Medical Center, Milwaukee, WI USA

**Keywords:** Quality of life, Ischemic heart disease, Questionnaire, MacNew

## Abstract

The aim of this study is to validate the Danish, Norwegian and Swedish versions of the self-administered MacNew Heart Disease Health-related Quality of Life questionnaire in patients with ischemic heart disease. The MacNew questionnaire, the Short Form SF-36, and the Hospital Anxiety and Depression Scale were completed at baseline by 976 patients (Denmark n = 353, Norway n = 328, Sweden n = 295) with a diagnosis of angina (n = 335), myocardial infarction (n = 352), or heart failure (n = 289). Each language version of the MacNew satisfied reliability criteria with Cronbach’s α values for the total group data (0.90–0.94) as well as the diagnostic group data (0.91–0.96). The test–retest correlations exceeded the criteria for group comparison (r ≥ 0.70) in Danish and Norwegian patients. The multidimensionality of the MacNew was confirmed although the original three-factor solution did not fully meet the criteria for good fit. Convergent and discriminative validity were confirmed in each language and diagnosis group with the exception of discriminative validity in Swedish angina patients. The psychometric properties of the Danish, Norwegian, and Swedish versions of the MacNew are largely confirmed. The MacNew can be recommended as a specific instrument for assessing and evaluating HRQL in Danish, Norwegian, and Swedish patients with angina, MI, and heart failure. However, the MacNew factor structure needs to be revisited in future studies.

## Background

Outcomes of existing and new therapies have been focused traditionally on mortality and morbidity. However, patient health status has been shown to be predictive of various health outcomes including cardiovascular events, hospitalizations, and healthcare cost (Rumsfeld et al. 2013). Patient reported health status is an important cardiovascular health outcome that includes three domains: symptom burden, functional status, and health related quality of life (HRQL; Rumsfeld et al. 2013).

Agencies such as the European Agency for the Evaluation of Medicinal Products (2005) and the US Food and Drug Administration (2009) have recommended the use of patient-reported treatment outcome measures such as HRQL in relevant research studies as well as in clinical care. The two basic formats for HRQL questionnaires—generic and disease-specific instruments—are designed for different purposes. General health survey researchers use generic questionnaires to assess a wide range of health states (Testa and Simonson 1996); specific HRQL questionnaires, with a focus on disease-relevant issues, are appropriate outcome measures in therapeutic intervention trials (Testa and Simonson 1996) as well as routine clinical care (Velikova et al. 2004; Cella et al. 1993). Marked health-status deficits, including poor HRQL, are commonly seen in patients with ischemic heart disease (IHD) and treatments such as medications, invasive interventions, or rehabilitation, with common therapeutic goals that include symptom management and improvement of patient HRQL, are frequently used in patients with different IHD diagnoses (Krumholz et al. 2005).

The self-administered MacNew Heart Disease HRQL questionnaire (MacNew), with an item stem referring to “your heart problem”, is a modification of the interviewer-administered Quality of Life after Myocardial Infarction questionnaire which was validated originally in English-speaking patients with MI (Höfer et al. 2004). There are 38 language versions of the MacNew with validation studies in patients with MI in 13 languages (n > 4,000), in patients with angina in 12 languages (n > 1,800), and in patients with heart failure in 11 languages (n > 550); all translated and validated versions of MacNew can be accessed at MacNew.org (Höfer et al. 2012). The MacNew has been validated in a small number of patients with IHD in Norway but without consideration of the specific IHD diagnosis (Hiller et al. 2010) and has not been validated in Danish or Swedish patients with IHD.

The objective of this study is to report on the psychometric properties of the MacNew in Danish, Norwegian, and Swedish patients with IHD and in each of the three major IHD diagnoses of angina, MI, or heart failure for the total group.

## Methods

### Patients

Patients with IHD from Denmark, Norway, and Sweden were recruited between 2003 and 2010 as part of the international HeartQoL Project conducted in 22 different countries (Oldridge et al. 2005). A convenience sample of patients ≥18 years, without a serious psychiatric disorder or active substance abuse, and who the referring physician considered able to complete the self-administered battery of HRQL instruments were eligible if they were being treated for: (A) angina (Canadian Cardiovascular Society class II, III or IV) with an objective measure of IHD (e.g., previous MI, exercise testing, echocardiogram, nuclear imaging or angiography); or for (B) a MI diagnosed at least 4 weeks and <6 months previously; or for (C) ischemic heart failure (New York Heart Association Class II, III or IV), with evidence of left ventricular dysfunction (ejection fraction ≤40 % by invasive or non-invasive testing), and an objective measure of IHD (e.g., previous MI, exercise testing, echocardiogram, nuclear imaging or angiography). Each respective Institutional Review Board approved the project and all subjects provided informed consent.

### Patient-Reported Outcome Assessment

The referring physician provided routine diagnostic data and all patients completed a self-report sociodemographic and clinical questionnaire. The Short-Form 36 Health Survey (SF-36) (Ware 2000), the Hospital Anxiety and Depression Scale (HADS) (Bjelland et al. 2002), and the MacNew (Höfer et al. 2004) were administered at baseline and 2 weeks later to approximately 20 % of the patients for testing instrument reliability.

#### SF-36

The SF-36 (version 1.0) is a valid generic health survey consisting of 36 items with 8 subscales summarized in two component scales: a physical component summary (PCS, including four subscales) and a mental component summary (MCS, including four subscales) scale. The SF-36 has been extensively used internationally (Ware 2000; Ware and Kosinski 2005) and has been validated in Danish (Bjorner et al. 1998), Norwegian (Loge and Kaasa 1998), and Swedish (Sullivan and Karlsson 1998).

#### HADS

The HADS is a valid psychological screening instrument designed to detect symptoms of anxiety and depression. It has been extensively used internationally (Bjelland et al. 2002). The HADS has been validated in Danish (Bjelland et al. 2002), Norwegian (Haug et al. 2004), and Swedish (Lisspers et al. 1997) in clinical trials with scores ≥8 used to screen and classify patients with mild or greater symptoms of depression and anxiety (Bjelland et al. 2002).

#### MacNew

The MacNew is designed to assess patient’s feelings about how IHD affects daily functioning and contains 27 items with a global HRQL score and physical limitation (13-item) and emotional (14-item), and social function (13-item) subscales with a 2-week timeframe, with 12 items falling into more than one domain. Examples of the subscale items include the following: “How often during the last 2 weeks have you experienced chest pain while doing your day-to-day activities?” (physical function); “How often during the last 2 weeks have you felt worthless or inadequate” (emotional function), and “How often during the past 2 weeks have you felt unable to socialize because of your heart problem?” (social function). The MacNew items and subscales are scored from 1 (low HRQL) to 7 (high HRQL) and has been described in detail elsewhere (Höfer et al. 2004). The minimal important difference (MID) on the MacNew Global scale and each subscale is 0.50 points (Dixon et al. 2002). Face and content validity, interpretability, respondent, and administrative burden of the MacNew have been previously established (Höfer et al. 2004). Using forward–backward translation, the MacNew was translated into Danish, Norwegian, and Swedish as part of the international HeartQoL project (Oldridge et al. 2005).

## Statistical Analysis

Patient clinical, sociodemographic, and scale characteristics are described using frequencies, means, and standard deviation (SD). The conceptual model, reliability, and validity of the MacNew were assessed (Scientific Advisory Committee of Medical Outcomes Trust 2002).

### Floor and Ceiling Effects

Floor effects occurred when patients scored at the lowest MacNew HRQL score (score = 1) and ceiling effects occurred when patients scored at the highest HRQL score (score = 7). The presence of a floor effect can indicate instrument sensitivity in detecting worsening health status, while ceiling effects indicate less sensitivity in detecting significant health improvements (Hays et al. 1998).

### Reliability

The reliability of the MacNew was evaluated by examining its internal consistency (Cronbach’s α); test–retest reliability (14-day) was assessed in a subsample of approximately 20 % patients with the intraclass correlation coefficient (ICC). A value of ≥0.70 was considered the criterion value for group comparisons and ≥0.90 for individual comparisons (Scientific Advisory Committee of Medical Outcomes Trust 2002).

### Factor Analysis

A confirmatory factor analysis was carried out using AMOS 18 (Byrne 2001). Chi square statistics are dependent on sample size and therefore the following parameters were used to evaluate data fit as they are less sensitive to sample size: (1) Chi square degree of freedom (χ^2^/df < 5; Hu and Bentler 1999); (2) root mean square error of approximation (RMSEA < 0.06); and (3) comparative fit index (CFI > 0.95) (Hu and Bentler 1999). Measurement errors of the items were allowed to inter-correlate where appropriate.

### Validity

As a test of construct validity, we compared the correlation coefficients between the corresponding SF-36 and MacNew scales with the non-corresponding scales using Steiger’s test for comparing Pearson correlations coefficients (Steiger 1980). We hypothesized strong correlations (r > 0.50) between the SF-36 Health Survey PCS and MCS and the similar MacNew scale constructs and significantly lower correlations between dissimilar constructs.

The ‘known group’ method (Hays et al. 1998) was used to test the discriminant validity of the MacNew by means of analysis of variance including post hoc comparisons (Bonferoni adjustment) in six separate analyses. In the first two analyses, patients were divided equally into tertiles (low, medium, high) based on their respective SF-36 PCS and MCS scores. We hypothesized that those patients who reported low SF-36 PCS and MCS scores would have poorer HRQL than those patients who reported medium or high SF-36 scores. In the next two analyses, we hypothesized that patients who showed symptoms of anxiety or depression on the HADS (HADS cut-off scores, ≥8) would have poorer HRQL than those patients who did not have symptom of anxiety or depression; in the final analyses, we hypothesized that patients in CCS or NYHA class III/IV would have poorer HRQL than those patients who were in CCS or NYHA class II.

## Results

A total of 976 patients with IHD from Denmark (n = 353), Norway (n = 328), and Sweden (n = 295) were recruited. There were 335 patients (34.3 %) with angina (Denmark, n = 140; Norway, n = 103; Sweden, n = 92), 289 patients (29.6 %) with heart failure (Denmark, n = 100; Norway, n = 104; Sweden, n = 85), and 352 patients (36.1 %) with MI (Denmark, n = 113; Norway, n = 121; Sweden, n = 118). In the group as a whole, the mean age was 65.1 years and 76.9 % were male (Table 1). There were significant between-country patient characteristic differences on age, gender, CCS angina and NYHA heart failure functional class (Table 1). Patient-reported outcome scores are also given in Table 1.

**Table 1:** Age, gender, diagnosis, and functional class (*CCS* Canadian Cardiovascular Society, *NYHA* New York Heart Association), and mean (±SD) patient-reported outcome scores: MacNew Global and subscales, Short-form Health Survey (SF-36; *PCS* physical component summary scale, *MCS* mental component summary scale), and HADS in the total group in each country

	Total group (n = 976)	Denmark (n = 353)	Norway (n = 328)	Sweden (n = 295)	*p* value
*Characteristics*					
Age (years)	65.1 (±10)	64.8 (±11)	63.2 (±10)	67.3 (±10)	<0.001^a,b^
Gender (% male)	76.9	72.8	83.2	74.9	<0.001^a,c^
*Diagnosis*					
Angina	335 (34.3 %)	140 (39.6 %)	103 (31.4 %)	92 (31.2 %)	0.48^b,c^
CCS Class II*	211 (63.3 %)	99 (70.4 %)	64 (62.1 %)	49 (57.6 %)	0.004^b,c^
CCS Class III/IV*	73 (21.8 %)	8 (5.6 %)	35 (34.0 %)	30 (32.6 %)	0.001^b,c^
MI	352 (36.1 %)	113 (29.1 %)	121 (36.9 %)	118 (40.0 %)	0.105
HF	289 (29.6 %)	100 (28.3 %)	104 (31.7 %)	85 (25.8 %)	0.649
NYHA Class II*	151 (52.2 %)	51 (51.0 %)	53 (51.0 %)	47 (53.3 %)	0.772
NYHA Class III/IV*	107 (37.0 %)	24 (23.8 %)	49 (47.1 %)	34 (40.0 %)	0.001^a,b^
*Patient*-*reported outcome scores*					
MacNew					
Global	5.3 (±1.0)	5.4 (±1.0)	5.1 (±1.0)	5.4 (±1.0)	<0.01^a,c^
Physical	5.1 (±1.2)	5.2 (±1.2)	4.8 (±1.2)	5.3 (±1.2)	<0.001^a,c^
Emotional	5.4 (±1.0)	5.5 (±1.1)	5.3 (±1.0)	5.4 (±1.0)	NS
Social	5.4 (±1.1)	5.6 (±1.1)	5.1 (±1.1)	5.4 (±1.1)	<0.001^a,b,c^
SF-36					
PCS	40.0 (±10.2)	41.6 (±10.3)	37.8 (±10.4)	40.6 (±10.0)	<0.001^a,c^
MCS	49.2 (±10.3)	49.6 (±10.4)	49.4 (±10.0)	49.0 (±10.4)	NS
HADS					
Anxiety	5.5 (±3.6)	6.7 (±3.4)	4.5 (±3.6)	5.2 (±3.9)	<0.001^b,c^
Depression	4.1 (±3.3)	4.1 (±3.5)	3.9 (±3.1)	4.3 (±3.4)	NS

* Missing data due to coding error; a = Swedish versus Norwegian; b = Swedish versus Danish; c = Norwegian versus Danish; at *p* < 0.001

### Patient-Reported Outcome Scores

The mean MacNew, SF-36, and HADS scores are given in Table 1 for the total group and in Table 2 for each diagnosis.

**Table 2:** Mean (±SD) patient-reported outcome scores: MacNew Global and subscales, SF-36 (*PCS* physical component summary scale, *MCS* mental component summary scale), and HADS in the total group in each diagnosis (angina; *MI* myocardial infarction, *HF* heart failure)

	Angina (n = 335)	MI (n = 352)	HF (n = 289)	*p* value
MacNew				
Global	5.2 (±1.0)	5.5 (±0.9)	5.1 (±1.0)	<0.001^a,b,c^
Physical	5.1 (±1.1)	5.5 (±1.1)	4.7 (±1.2)	<0.001^a,b,c^
Emotional	5.3 (±1.1)	5.6 (±0.9)	5.3 (±1.1)	<0.001^a,b^
Social	5.4 (±1.1)	5.6 (±1.1)	5.1 (±1.2)	<0.001^b,c^
SF-36				
PCS	39.5 (±9.2)	43.5 (±9.9)	36.1 (±10.7)	<0.001^a,b,c^
MCS	48.9 (±10.8)	49.6 (±9.3)	49.0 (±10.9)	0.698
HADS				
Anxiety	6.3 (±3.7)	4.6 (±3.5)	5.6 (±3.8)	<0.001^a,b^
Depression	4.2 (±3.3)	3.6 (±3.1)	4.6 (±3.6)	<0.001^a.b^

^a^MI versus Angina; ^b^ MI versus HF; ^c^ Angina versus HF at *p* < 0.05

#### Patient-Reported Outcome Scores for the Total Group (Table 1)

The mean MacNew Global and subscale scores ranged from a low of 5.1 on the physical scale to 5.4 on both the emotional and social subscales. The SF-36 mean PCS score at 40.0 was 1 standard below the standardized mean of 50.0 with the mean MCS score almost the same as the standardized mean score at 49.2. The mean HADS anxiety and depression scores were below the HADS threshold of ≥8 for possible anxiety and depression.

#### Patient-Reported Outcome Scores by Country (Table 1)

The mean MacNew Global scores were 5.4 for both the Danish and Swedish patients and 5.1 for the Norwegian patients. The subscale scores were similar ranging from 5.2 to 5.6 for Danish patients, 4.8–5.1 for Norwegian patients and 5.3–5.4 for Swedish patients. The SF-36 PCS mean scores were 41.6 for the Danish patients and 40.6 for the Swedish patients; the Norwegian patients, at a score of 37.8 were >1 SD below the standardized mean of 50.0. The SF-MCS mean scores for the Danish patients (49.6), Norwegian patients (49.4) and Swedish patients (49.0) were all close to the standardized mean of 50.0. The mean HADS anxiety and depression scores were below the HADS threshold of ≥8 for all three countries.

#### Patient Reported Outcome Scores by Diagnosis (Table 2)

There were significant between-diagnosis differences in MacNew Global and each subscale scores (*p* < 0.001). In patients with MI, the MacNew Global and all subscale scores were always significantly higher than in patients with heart failure (*p* < 0.001). In addition MI patients scored significantly higher than patients with angina on the MacNew Global, physical, and emotional subscales (*p* < 0.001). Patients with angina reported higher MacNew Global, physical, and social subscale scores than patients with heart failure (*p* < 0.001). There were significant between-diagnosis differences in PCS scores but not in MCS scores. The PCS scores were higher in patients with MI than in patients with either angina or heart failure (*p* < 0.001) while patients with angina had higher PCS scores than patients with heart failure (*p* < 0.05). There were significant between-diagnosis differences in HADS anxiety and depression scores. MI patients reported lower scores for anxiety and depression, than patients with either angina or heart failure (*p* < 0.001).

### MacNew Psychometric Properties by Country

#### Floor and Ceiling Effects

There were no floor effects in the MacNew Global and subscale scores as reported by the patients in each country. Likewise no ceiling effects occurred on the MacNew Global scale scores although there were ceiling effects in the subscale scores for 1.1–4.8 % of Danish patients, 0.0–1.5 % of Norwegian patients, and 1.0–2.0 % of Swedish patients (Table 3).

**Table 3:** MacNew reliability by country and by diagnosis; internal consistency (Cronbach’s α) and test–retest reliability (ICC) for MacNew Global score and subscale scores

MacNew	Danish (n = 353)				Norwegian (n = 328)				Swedish (n = 295)			
	Global	Physical	Emotional	Social	Global	Physical	Emotional	Social	Global	Physical	Emotional	Social
*Country*												
Internal consistency	0.96	0.93	0.93	0.93	0.94	0.90	0.94	0.91	0.94	0.91	0.94	0.92
Test–retest reliability	0.82*	0.76*	0.89*	0.87*	0.90*	0.91*	0.88*	0.90*	0.68*	0.79*	0.62*	0.77*

	Angina (n = 335)				MI (n = 352)				Heart failure (n = 289)			
*Diagnosis*												
Internal consistency	0.96	0.91	0.94	0.93	0.95	0.91	0.92	0.92	0.95	0.91	0.94	0.91
Test–retest reliability	0.53*	0.52*	0.64*	0.64*	0.86*	0.95*	0.65*	0.89*	0.94*	0.93*	0.095*	0.91*

* *p* < 0.01

#### Reliability (Table 3)

(A) Internal consistency reliability: Cronbach’s α ranged from 0.94 to 0.96 on the Global scale for the total cohort of Danish, Norwegian, and Swedish patients and from 0.90 to 0.94 on the subscales.
(B) Test–retest reliability: The 14-day ICC was always significant and exceeded the population criteria of ≥0.70 on the Global and each subscale in Danish and Norwegian patients on the physical and social subscales in Swedish patients.

#### Factor Analysis

Our findings in general support the multidimensionality of the MacNew (Höfer et al. 2004, 2012; Hiller et al. 2010; Vandereyt et al. 2012). However, after allowing item measurement errors to inter-correlate where appropriate, the confirmatory three-factor model did not fully support the combined data (Denmark: χ^2^/df = 3.37; CFI = 0.90 RMSEA = 0.08; explained variance = 48.5 %, Norway: χ^2^/df = 3.72; CFI = 0.88; RMSEA = 0.09; explained variance = 50.9 %, Sweden: χ^2^/df = 2.99; CFI = 0.90; RMSEA = 0.08; explained variance = 49.6 %). The interclass correlation coefficients ranged from 0.66 to 0.81 for the Danish MacNew, from 0.66 to 0.83 for the Norwegian MacNew, and from 0.68 to 0.84 for the Swedish MacNew. In the original MacNew factor analysis based on patients with MI, and since then also in other language validation studies (Höfer et al. 2004), a high proportion of social subscale items (10 of 13) cross-loaded with more than one subscale with factor loadings ≥0.40. In the present analysis, social subscale items cross-loaded (n = 7 for Denmark; n = 3 for both Norway and Sweden) with either the physical or emotional subscales with loadings ≥0.40. Item #27 about sexual activity, which was not part of the original factor analysis (Höfer et al. 2004), fitted best with the social subscale.

#### Convergent Validity (Table 4)

Convergent validity of the MacNew physical and emotional subscales with the SF-36 PCS and MCS was confirmed in each language with correlations always ≥0.70 and statistically significant at (*p* < 0.001). The correlations between dissimilar constructs (e.g., MacNew physical x SF-36 MCS) were significantly lower (*p* < 0.001) than between similar constructs.

**Table 4:** MacNew convergent validity by country and total group by diagnosis: MacNew physical and emotional subscales and SF-36 component summary scales—physical (PCS) and mental (MCS)

MacNew	Danish (n = 353)		Norwegian (n = 328)		Swedish (n = 295)	
	Physical	Emotional	Physical	Emotional	Physical	Emotional
*Country*						
SF-36 PCS	0.78*	0.526*	0.70*	0.44*	0.70*	0.37*
SF-36 MCS	0.58*	0.781*	0.50*	0.71*	0.45*	0.71*

	Angina (n = 335)		MI (n = 352)		Heart failure (n = 289)	
*Diagnosis*						
SF-36 PCS	0.73*	0.56*	0.72*	0.21*	0.69*	0.42*
SF-36 MCS	0.46*	0.78*	0.45*	0.70*	0.50*	0.73*

* 1-sided *p* value <0.001

#### Discriminative Validity (Table 5)

Discriminative validity for the MacNew was confirmed statistically and either met or exceeded the MacNew MID for differences in MacNew scores between patients in the low SF-36 PCS and MCS groups when compared to both the medium and high HRQL groups and for those with and without anxiety or depression in each country. The differences in MacNew scores between patients with CCS Class II and CCS Class III/IV angina were statistically significant only for the social subscale in Danish patients and for the Global, physical, and social scales in Norwegian patients. On the other hand, the differences in MacNew scores between patients with CCS Class II and Class III/IV angina met or exceeded the MID on all scales in Danish and Norwegian patients but not in the Swedish patients. The differences between patients with NYHA Class II and Class III/IV heart failure were statistically significant and met or exceeded the MID in each country for all MacNew scores.

**Table 5:** MacNew discriminant validity by country: MacNew Global score and subscale scores (mean ± SD) by SF-36 component summary scales—physical (PCS) and mental (MCS) (tertiles) and HADS (symptom present, score 8+, symptom absent <8) and functional class (*CCS* Canadian Cardiovascular Society, *NYHA* New York Heart Association)

MacNew	Danish (n = 353)				Norwegian (n = 328)				Swedish (n = 295)			
	Global	Physical	Emotional	Social	Global	Physical	Emotional	Social	Global	Physical	Emotional	Social
*SF*-*36 PCS*												
Low HRQL	4.5 ± 0.9	4.1 ± 1.0	4.7 ± 1.1	4.6 ± 1.0	4.6 ± 0.9	4.1 ± 0.9	4.9 ± 0.9	4.5 ± 1.0	4.6 ± 0.9	4.3 ± 0.9	4.9 ± 1.0	4.9 ± 1.0
Medium HRQL	5.3 ± 0.8	5.2 ± 0.8	5.4 ± 1.0	5.6 ± 0.9	5.2 ± 0.8	5.0 ± 0.8	5.4 ± 0.9	5.2 ± 0.9	5.4 ± 0.8	5.3 ± 0.8	5.5 ± 0.9	5.5 ± 0.8
High HRQL	6.1 ± 0.7	6.1 ± 0.7	6.0 ± 0.8	6.4 ± 0.7	6.0 ± 0.6	6.0 ± 0.7	6.0 ± 0.9	6.1 ± 0.7	6.1 ± 0.8	6.1 ± 0.8	5.8 ± 0.8	6.1 ± 0.8
*p* value	<0.001	<0.001	<0.001	<0.001	<0.001	<0.001	<0.001	<0.001	<0.001	<0.001	<0.001	<0.001
*SF*-*36 MCS*												
Low HRQL	4.5 ± 0.9	4.4 ± 0.9	4.5 ± 0.9	4.8 ± 0.9	4.4 ± 0.9	4.2 ± 1.0	4.4 ± 1.0	4.4 ± 1.0	4.7 ± 0.9	4.6 ± 1.1	4.6 ± 0.9	4.7 ± 1.0
Medium HRQL	5.5 ± 0.9	5.3 ± 0.9	5.6 ± 0.9	5.7 ± 0.9	5.1 ± 0.8	5.0 ± 1.0	5.5 ± 0.0	5.3 ± 0.9	5.5 ± 0.8	5.3 ± 0.9	5.5 ± 0.7	5.5 ± 0.9
High HRQL	6.2 ± 0.9	6.0 ± 0.9	6.3 ± 0.9	6.4 ± 0.9	5.7 ± 0.8	5.4 ± 1.0	6.0 ± 0.6	5.7 ± 0.9	6.0 ± 0.6	5.8 ± 0.9	6.1 ± 0.6	6.1 ± 0.8
*p* value	<0.001	<0.001	<0.001	<0.001	<0.001	<0.001	<0.001	<0.001	<0.001	<0.001	<0.001	<0.001
*Anxiety*												
No	5.9 ± 0.9	5.6 ± 1.2	6.0 ± 0.8	6.0 ± 1.0	5.4 ± 0.9	5.0 ± 1.1	5.6 ± 0.8	5.3 ± 1.0	5.6 ± 0.9	5.4 ± 1.1	5.8 ± 0.8	5.6 ± 1.1
Yes	4.7 ± 0.9	4.7 ± 1.1	4.7 ± 0.9	5.0 ± 1.1	4.1 ± 0.9	4.0 ± 1.1	4.0 ± 1.0	4.2 ± 1.0	4.6 ± 0.8	4.7 ± 1.1	4.5 ± 0.8	4.8 ± 1.0
*p* value	<0.001	<0.001	<0.001	<0.001	<0.001	<0.001	<0.001	<0.001	<0.001	<0.001	<0.001	<0.001
*Depression*												
No	5.6 ± 0.9	5.4 ± 1.1	5.7 ± 0.9	5.9 ± 1.0	5.3 ± 0.9	5.0 ± 1.1	5.6 ± 0.9	5.3 ± 1.0	5.6 ± 0.9	5.5 ± 1.0	5.7 ± 0.8	5.6 ± 1.0
Yes	4.1 ± 0.9	4.0 ± 1.1	4.0 ± 1.0	4.3 ± 1.2	3.8 ± 0.7	3.6 ± 1.0	3.8 ± 0.8	3.8 ± 0.9	4.3 ± 0.8	4.3 ± 1.0	4.3 ± 0.8	4.4 ± 1.0
*p* value	<0.001	<0.001	<0.001	<0.001	<0.001	<0.001	<0.001	<0.001	<0.001	<0.001	<0.001	<0.001
*CCS Class*												
CCS II	5.3 ± 1.0	5.2 ± 1.1	5.3 ± 1.1	5.6 ± 1.0	5.1 ± 1.0	5.5 ± 0.9	5.8 ± 0.9	5.7 ± 0.8	5.5 ± 0.9	5.4 ± 1.1	5.6 ± 1.0	5.6 ± 1.1
CCS III/IV	4.6 ± 1.3	4.7 ± 1.1	4.5 ± 1.5	4.8 ± 1.2	4.6 ± 1.1	4.7 ± 1.1	5.3 ± 0.8	5.0 ± 1.0	5.1 ± 1.3	5.2 ± 1.4	5.2 ± 1.3	5.2 ± 1.4
*p* value	ns	ns	ns	0.049	0.014	0.004	ns	<0.001	ns	ns	ns	ns
*NYHA Class*												
NYHA II	5.5 ± 0.9	5.3 ± 1.1	5.7 ± 1.0	5.6 ± 1.0	5.3 ± 0.0	4.9 ± 1.0	5.5 ± 0.0	5.3 ± 1.1	5.2 ± 0.9	5.1 ± 1.0	5.4 ± 1.0	5.3 ± 1.0
NYHA III/IV	4.8 ± 1.1	4.2 ± 1.1	5.1 ± 1.2	4.8 ± 1.1	4.5 ± 1.0	4.1 ± 1.1	4.8 ± 1.0	4.3 ± 1.2	4.6 ± 0.8	4.2 ± 1.0	4.9 ± 1.0	4.5 ± 1.1
*p* value	0.002	<0.001	0.018	<0.001	<0.001	<0.001	0.008	<0.001	<0.001	<0.001	0.019	<0.001

### MacNew Psychometric Properties by Diagnosis

#### Floor and Ceiling Effects

There were no floor effects in the MacNew Global scale and subscale scores as reported by patients in each diagnosis. However, ceiling effects occurred in the MacNew Global scale and subscale scores for 0.0–4.3 % of angina patients, 0.0–2.0 % of heart failure patients, and 0.0–3.8 % of patients with MI.

#### Reliability (Table 3)

(A) Internal consistency reliability: Cronbach’s α was remarkably similar across diagnoses, ranging from 0.91 to 0.96 for the Global scale and subscales.
(B) Test–retest reliability: The 14-day ICC for the Global scale and each subscale was significant (*p* < 0.01) in each diagnosis. In patients with angina the ICC ranged from 0.53 to 0.64; in patients with MI from 0.65 to 0.95; and in patients with heart failure the ICC values were more consistent, ranging from 0.91 to 0.95.

#### Factor Analysis

The results for the confirmatory three-factor model per diagnosis were partly confirmed; Angina: χ^2^/df = 2.98; CFI = 0.92 RMSEA = 0.06; explained variance = 51.2 %; MI: χ^2^/df = 2.76; CFI = 0.93; RMSEA = 0.07; explained variance = 52.5 %; HF: χ^2^/df = 2.79; CFI = 0.90; RMSEA = 0.07; explained variance = 50.3 %.

#### Convergent Validity (Table 4)

Convergent validity of the MacNew physical and emotional subscales and the SF-36 PCS and MCS scales was confirmed in each diagnosis with correlations always ≥0.69. The correlation coefficients between dissimilar scales (e.g., MacNew physical × SF-36 MCS) were all significantly lower than the correlation coefficients between similar scales (*p* < 0.001).

#### Discriminative Validity (Table 6)

Statistical significance was demonstrated in every SF-36 PCS and MCS, anxiety, and depression MacNew score comparison in each diagnosis and by CCS classification in patients with angina and NYHA classification in patients with heart failure. In addition, the MacNew MID was met or exceeded in all SF-36 PCS and MCS, all anxiety and depression, and all NYHA and CCS comparisons with the only exception being the Global MacNew score in patients with angina.

**Table 6:** Discriminant validity by diagnosis: MacNew Global score and subscale scores (mean ± SD) by SF-36 component summary scales [physical (PCS) and mental (MCS) (tertiles)] and HADS (symptom present, score 8 + , symptom absent < 8) and functional class (*CCS* Canadian Cardiovascular Society, *NYHA* New York Heart Association)

MacNew	Angina (n = 335)				MI (n = 352)				Heart failure (n = 289)			
	Global	Physical	Emotional	Social	Global	Physical	Emotional	Social	Global	Physical	Emotional	Social
*SF*-*36 PCS*												
Low HRQL	4.4 ± 0.9	4.1 ± 0.9	4.6 ± 1.0	4.5 ± 1.0	4.6 ± 0.8	4.2 ± 1.0	4.9 ± 0.9	4.6 ± 1.0	4.6 ± 1.0	4.1 ± 1.0	4.9 ± 1.1	4.5 ± 1.1
Medium HRQL	5.3 ± 0.8	5.2 ± 0.8	5.4 ± 1.0	5.5 ± 0.9	5.4 ± 0.8	5.3 ± 0.8	5.4 ± 0.9	5.4 ± 0.9	5.2 ± 0.7	5.0 ± 0.8	5.4 ± 0.9	5.3 ± 0.8
High HRQL	6.1 ± 0.7	6.1 ± 0.7	6.0 ± 0.8	6.0 ± 0.7	6.0 ± 0.7	6.1 ± 0.7	5.9 ± 0.8	6.1 ± 0.8	6.0 ± 0.6	5.9 ± 0.8	6.0 ± 0.7	6.2 ± 0.7
*p* value	<0.001	<0.001	<0.001	<0.001	<0.001	<0.001	<0.001	<0.001	<0.001	<0.001	<0.001	<0.001
*SF*-*36 MCS*												
Low HRQL	4.3 ± 0.8	4.3 ± 1.0	4.3 ± 0.9	4.6 ± 1.0	4.8 ± 0.8	4.8 ± 1.0	4.8 ± 0.8	4.9 ± 1.0	4.3 ± 0.8	4.0 ± 1.0	4.4 ± 1.0	4.3 ± 1.0
Medium HRQL	5.4 ± 0.8	5.2 ± 1.0	5.5 ± 0.8	5.6 ± 0.9	5.6 ± 0.8	5.5 ± 1.0	5.7 ± 0.8	5.7 ± 0.9	5.3 ± 0.8	4.9 ± 1.0	5.5 ± 0.8	5.2 ± 1.0
High HRQL	6.0 ± 0.6	5.8 ± 0.8	6.2 ± 0.6	6.2 ± 0.7	6.1 ± 0.6	6.1 ± 0.8	6.2 ± 0.6	6.2 ± 0.8	5.8 ± 0.7	5.3 ± 1.0	6.1 ± 0.6	5.8 ± 1.0
*p* value	<0.001	<0.001	<0.001	<0.001	<0.001	<0.001	<0.001	<0.001	<0.001	<0.001	<0.001	<0.001
*Anxiety*												
No	5.5 ± 0.9	5.3 ± 1.1	5.7 ± 0.9	5.5 ± 1.0	5.6 ± 0.8	5.5 ± 1.0	5.8 ± 0.7	5.6 ± 1.0	5.2 ± 0.8	4.8 ± 1.1	5.6 ± 0.8	5.1 ± 1.1
Yes	4.3 ± 0.9	4.3 ± 1.1	4.2 ± 0.8	4.5 ± 1.1	4.8 ± 0.7	4.9 ± 0.9	4.6 ± 0.8	4.9 ± 0.8	4.2 ± 1.0	4.1 ± 1.2	4.1 ± 1.0	4.3 ± 1.2
*p* value	<0.001	<0.001	<0.001	<0.001	<0.001	<0.001	<0.001	<0.001	<0.001	<0.001	<0.001	<0.001
*Depression*												
No	5.4 ± 1.0	5.2 ± 1.1	5.5 ± 1.0	5.5 ± 1.1	5.6 ± 0.7	5.5 ± 0.9	5.7 ± 0.8	5.6 ± 0.9	5.3 ± 0.8	4.9 ± 1.1	5.6 ± 0.8	5.2 ± 1.0
Yes	4.1 ± 0.7	4.1 ± 1.0	4.1 ± 0.8	4.1 ± 0.7	4.3 ± 0.7	4.2 ± 1.1	4.3 ± 0.7	4.3 ± 1.0	4.0 ± 0.9	3.8 ± 1.1	3.9 ± 0.9	4.0 ± 1.1
*p* value	<0.001	<0.001	<0.001	<0.001	<0.001	<0.001	<0.001	<0.001	<0.001	<0.001	<0.001	<0.001
*CCS Class*												
CCS II	5.3 ± 1.0	5.2 ± 1.1	5.4 ± 1.0	5.6 ± 1.0								
CCS III/IV	4.9 ± 1.2	4.6 ± 1.3	5.0 ± 1.3	4.9 ± 1.3								
*p* value	<0.001	<0.001	<0.008	<0.0.001								
*NYHA Class*												
NYHA II									5.4 ± 0.9	5.1 ± 1.1	5.6 ± 1.0	5.4 ± 1.0
NYHA III/IV									4.6 ± 1.0	4.2 ± 1.0	4.9 ± 1.1	4.5 ± 1.1
*p* value									<0.0001	<0.0001	<0.0001	<0.0001

## Discussion

The psychometric properties of reliability and validity for the MacNew Heart Disease Health-related Quality of Life questionnaire, with probes that are relevant to patients with angina, MI, or heart failure, are largely supported in Danish, Norwegian, and Swedish patients with the exception of the ICC values in patients with angina and the partial confirmation of the 3-factor solution. These observations on score distributions, internal consistency reliability, and both convergent and discriminative validity in these Scandinavian patients with IHD essentially confirm previous results reported in MacNew validation studies in other languages (Höfer et al. 2004, 2012; Vandereyt et al. 2012). The overall floor and ceiling effect findings are similar to previous international MacNew validation studies (Höfer et al. 2004, 2012; Vandereyt et al. 2012) with no floor effects and minor ceiling effects in the scores by country or diagnosis. This indicates good sensitivity for detecting statistically significant changes in both directions (improvement or deterioration) of HRQL in Scandinavian patients.

As Rumsfeld and colleagues have pointed out, “patient-reported health status measures reflect how an individual views and adapts to his or her symptom burden, functional limitations, and prognosis, as well as how patients perceive their overall health” (Rumsfeld et al. 2013). As such, patient-reported health status measures like the MacNew HRQL questionnaire have the potential to support quality clinical care, to serve as a foundation for shared medical decision making with patients, to evaluate treatment effectiveness by monitoring the impact of interventions, and to identify patients for prognostic discussions. The patient’s input may be important for risk adjustment and for targeting healthcare resources such as disease management to those with the largest health deficits. Inclusion of patient-reported health status in national surveillance of heart disease will ensure that cardiovascular health, as reflected in these patient health status measures, is accounted for when making health policy decisions. However, all of these objectives for HRQL assessment in clinical care, research, and policy decision-making require psychometrically valid instruments with interpretable scores to contribute usefully to clinical decision-making.

The interpretation of HRQL scores is important for clinicians who need to have a sense of where their patient falls in terms of normative data when making clinical decisions. The overall scores for the Global and each MacNew subscale are consistent with previous international observations for the group as a whole and for each clinical diagnosis where the highest HRQL is seen in patients with MI and the lowest HRQL in patients with heart failure (Höfer et al. 2004, 2012; Maes et al. 2008; Vandereyt et al. 2012). The MID is the smallest change in HRQL that patients perceive as important would help clinicians and patients to consider a change in management (Schunemann et al. 2005) and has been established as a 0.50 point change or difference on the 7-point MacNew scale (Dixon et al. 2002). Of the 12 between-country comparisons on the MacNew Global and each subscale score, two meet or exceed the MID; these are (1) between and Swedish (mean = 5.3) and Norwegian patients (mean = 4.8) on the physical subscale and (2) between Danish (mean = 5.6) and Norwegian patients (5.1) on the social subscale. Unfortunately there is little information on cross-country HRQL comparisons in patients with IHD; HRQL was assessed six months after discharge from hospital in 22 European countries in the EuroAspire III survey of patients with IHD but Denmark, Norway, and Sweden did not participate in the study (De Smedt et al. 2013). Therefore we are able to provide for the first time country and disease specific results, which will allow clinicians to put their individual patient’s score into perspective and sets a standard for future country specific analyses.

The Danish, Norwegian, and Swedish versions of the MacNew have adequate internal consistency reliability with Cronbach’s α values for the country data (0.90–0.94) and for the diagnostic data (0.91–0.96) allowing for comparison of individual patients as well as group level data and substantiating previous work (Höfer et al. 2012; Vandereyt et al. 2012). The test–retest correlations exceeded the criterion for group comparison (r ≥ 0.70) indicating acceptable reproducibility of the MacNew in the Danish and Norwegian patients; on the other hand, the criterion value was not met in Swedish patients on the emotional subscale (r = 0.62) which, in turn, reflects on the ICC value for the Global MacNew score in Swedish patients (r = 0.68). While ICC values were consistent in patients with heart failure and MI, there was an issue with the ICC values for patients with angina (r = 0.53–0.64), which could be linked specifically to the Swedish sampling of patients with angina who may not have been in a clinically stable phase when the MacNew was administered over time. Overall, this suggests that the MacNew is reliable and reproducible in Danish and Norwegian patients with angina, MI, and heart failure, and in Swedish patients with MI, and heart failure.

Although the multidimensionality of the MacNew was confirmed, the three-factor solution for the MacNew did not fully meet the criteria set for confirmation in the present study. While the Chi square data met the criterion of <5, the CFI data and the RMSEA did not meet the criteria of >0.95 and <0.06, respectively (Byrne 2001). Previous publications have highlighted similar issues with the MacNew factor structure (Dempster et al. 2004). It has been suggested that these issues may be due to sample size problems and distributional misspecifications that together increase the reliance on alternative fit indices (Dempster et al. 2004). However, this needs to be revisited in future studies.

There is consistent evidence of convergent validity in each language version of the MacNew and within each diagnosis. Convergent validity of the MacNew physical and emotional subscales with the SF-36 PCS and MCS for the total group in each language was demonstrated although, as observed in other studies (Höfer et al. 2004, 2012; Vandereyt et al. 2012), the correlations for dissimilar constructs were higher than expected. It has previously been suggested that this is most likely a result of the differing perspectives of the MacNew and SF-36 questionnaires (Dempster et al. 2004). As reflected by their respective probes, the MacNew focuses on how patients feel after their cardiac event and perceive their quality of life while the SF-36 focuses on how patients do, i.e., perform, after a cardiac event. In other words, the high correlations between the physical MacNew and SF-36 MCS scales may reflect the perception of limitations in the MacNew rather than “actual” performance limitations as in the SF-36.

Discriminative validity was largely confirmed in the three languages and in the diagnosis groups. Patients with heart failure always had poorer MacNew Global and each subscale scores than patients with MI except on the SF-36 MCS score. Patients who fell into the low SF-36 MCS or PCS HRQL groups, patients who met the HADS criteria for symptoms of anxiety or depression, and patients who had NYHA Class III/IV heart failure consistently reported lower scores on each MacNew scale which were both statistically significant and clinically important when compared to patients who fell into either the medium or high HRQL SF-36 HRQL groups, patients who were neither showing symptoms of anxiety nor depression, and patients who had NYHA Class II heart failure. On the other hand, CCS angina differences were not as consistent. When comparing CCS Class II and CCS Class III/IV angina patients within each country, all MacNew score differences in Danish and Norwegian patients met or exceeded the MID with four of the eight comparisons statistically significant: the social subscale in Danish patients and the Global, physical, and social scales in Norwegian patients. None of the MacNew score differences by CCS Class were statistically significant or met the MID in Swedish patients. An important question is whether the substantial differences in the MacNew scores observed between patients with Class II and Class III/IV angina matter from a clinical perspective? In this case, the differences in both Danish and Norwegian patients with Class II and Class III/IV angina suggest important differences from a clinical perspective. This is in contrast to the differences in Swedish patients which did not meet the MID which again might be due to the sampling of the Swedish angina patients.

The parent HeartQoL Project was designed as a cross-sectional survey and recruited convenience samples of patients at each site. As a result, limitations include our inability to generate and report responsiveness statistics for the MacNew in these patients. The different strategies used to recruit patients across the three different countries as part of the international HeartQoL Project (Oldridge et al. 2005) could have resulted in the specific HRQL differences, e.g., the discriminative validity difference in patients with angina. Further, the patients recruited were also predominantly male (72.8–83.2 %) limiting the interpretation of the validity of the MacNew in female patients. In addition, the angina and heart failure diagnosis comparisons were somewhat limited with a relatively high percentage of CCS (24.0 %) and NYHA (25.7 %) classification details missing from the Danish data, which occurred as a result of a recording error.

## Conclusions

The psychometric properties of the Danish, Norwegian, and Swedish versions of the MacNew are largely confirmed. The MacNew is a reliable and valid measure providing a comprehensive assessment of HRQL among these patients with IHD and a specific diagnosis of angina, MI, or heart failure with some exceptions, particularly in Swedish angina patients. The MacNew can be recommended as a disease-specific instrument for assessing and evaluating HRQL in Danish, Norwegian, and Swedish patients with angina, MI, and heart failure. This study contributes to the growing international evidence supporting the MacNew as a core disease-specific HLQL measure for patients with IHD.
